# Synergistic effects of nitrogen metabolites on auxin regulating plant growth and development

**DOI:** 10.3389/fpls.2022.1098787

**Published:** 2022-12-20

**Authors:** Yu-Fan Fu, Xin-Yue Yang, Zhong-Wei Zhang, Shu Yuan

**Affiliations:** College of Resources, Sichuan Agricultural University, Chengdu, China

**Keywords:** nitrogen metabolites, ammonium, nitrate reductase, nitric oxide, tryptophan aminotransferase, auxin signaling and transport

## Abstract

Nitrogen is one of the important nutrients required for plant growth and development. There is increasing evidences that almost all types of nitrogen metabolites affect, at least to some extent, auxin content and/or signaling in plants, which in turn affects seed germination, plant root elongation, gravitropism, leaf expansion and floral transition. This opinion focuses on the roles of nitrogen metabolites, ${\text{NO}}_{3}^{-}$ , ${\text{NH}}_{4}^{+}$ , tryptophan and NO and their synergistic effects with auxin on plant growth and development. Nitrate reductase (NR) converts nitrate into nitrite, and was roughly positive-correlated with the root auxin level, suggesting a crosstalk between nitrate signaling and auxin signaling. Abscisic Acid Responsive Element Binding Factor 3 (AFB3) and Tryptophan Aminotransferase of Arabidopsis 1 (TAA1) are also the key enzymes involved in nitrogen metabolite-regulated auxin biosynthesis. Recent advances in the crosstalk among ${\text{NO}}_{3}^{-}$ , ${\text{NH}}_{4}^{+}$ , tryptophan and NO in regulation to NR, AFB3 and TAA1 are also summarized.

## Introduction

Nitrogen is one of the important nutrients required by plant growth and development. Plant roots can access nitrogen (N) in various forms which include organic compounds. The primary N forms root absorbs are ammonium ( ${\text{NH}}_{4}^{+}$ ), nitrate ( ${\text{NO}}_{3}^{-}$ ), and amino acids. Typically, the most plentiful source of N is nitrate (Miller et al., 2007; Kant et al., 2011).

The cytosolic enzyme nitrate reductase (NR) first converts nitrate inside the cell to nitrite, which is a rate-limiting step in the assimilation pathway. The nitrite is transported into the chloroplast (Fernandez and Galvan, 2007). Plastid nitrite reductase (NiR) catalyzes the conversion of nitrite to ammonium, which is then absorbed into carbon skeletons by producing glutamate *via* the glutamine synthetase/glutamine oxoglutarate aminotransferase (GS/GOGAT) cycle (Sanz-Luque et al., 2015). As a result, nitrate assimilation occurs *via* a rather straightforward linear process that includes two transport phases (nitrate and nitrite transport) and two reduction steps (involving NR and NiR) (Chamizo-Ampudia et al., 2017).

## Roles of ${\text{NO}}_{3}^{-}$ on plant growth and development

${\text{NO}}_{3}^{-}$ works as a signaling molecule to influence plant growth and development, as well as serving as a main N source for plants (Miller et al., 2007). This leads to a theory that plant cells needed an availability sensor for ${\text{NO}}_{3}^{-}$ . Local ${\text{NO}}_{3}^{-}$ availability regulates the expression of nitrate assimilation genes (Krapp et al., 2014), breaks seed dormancy (Alboresi et al., 2005; Liu et al., 2021), controls leaf morphogenesis (Yang et al., 2022), stimulates the formation and extension of lateral roots (Forde and Walch-Liu, 2009; Chen et al., 2018; Contreras-López et al., 2022) and postpones flowering (Yuan et al., 2016; Sanagi et al., 2021; Ye et al., 2021; Zhang S. et al., 2021). Lack of nitrate also affects tomato fruit yield and quality (Belfry et al., 2017) and maize stem internodes development (Peng et al., 2013).

The nitrate transporter NRT1.1 is a dual-affinity nitrate transceptor controlling the primary nitrate responses (nitrate signaling), in which expressions of nitrate assimilation genes and nitrate transporter genes are induced rapidly by nitrate treatments (Ho et al., 2009). NRT1.1 facilitates not only nitrate uptake but also auxin transport. Nitrate treatments repress NRT1.1-mediated auxin uptake, indicating that the nitrate signaling *via* NRT1.1 is correlated with a regulation of auxin transport (Krouk et al., 2010). Another report found that expression of Abscisic Acid Responsive Element Binding Factor 3 (AFB3) depends on the nitrate-transport function of NRT1.1 (Vidal et al., 2014).

Recently, the NIN-like protein (NLP) transcription factor NLP7 has also been suggested as a nitrate sensor (Liu et al., 2022). NLP7 is a crucial nitrate signaling regulator that binds directly to the *TAR2* (Tryptophan Aminotransferase Related 2) promoter and activates its expression to sustain auxin signaling in the root primordia (Liu et al., 2017; Zhang T. T, et al., 2021).

Nitrate inhibits Ferredoxin-NADP^+^-Oxidoreductase (*FNR1*) expression, therefore causing declines in NADPH/NADP^+^ and ATP/AMP ratios, which in-turn promotes adenosine monophosphate-activated protein kinase (AMPK) activities and modulates their nuclear abundance (Yuan et al., 2016). KIN10 phosphorylates NLP7 to induce its cytoplasmic retention and the subsequent degradation, therefore repressing nitrate-regulated gene expression and inhibiting growth (Wang et al., 2021).

The *nia1*/*nia2* (nitrate reductase) double mutant showed significantly low transcription levels of auxin biosynthesis/signaling genes and was insensitive to nitrogen changes. NR activity was roughly positive-correlated with the root auxin content, and there should be a crosstalk between nitrate signaling and auxin accumulation (Fu et al., 2020).

Another interesting study indicated that in the *nia1*/*nia2* double mutant, the auxin signaling gene *AFB3* expression was increased by the nitrate with the max level at 1 hour, as well as in the wild-type seedlings. Nevertheless contrasting to the wild-type seedlings, the *AFB3* mRNA did not decrease in the *nia1/nia2* double mutant after 1 hour (Vidal et al., 2010). The authors concluded that NR may regulate both auxin biosynthesis and auxin signaling, and some nitrate metabolite downstream of NR may control *AFB3* expression indirectly (Vidal et al., 2010; Vidal et al., 2013).

**Figure 1:**
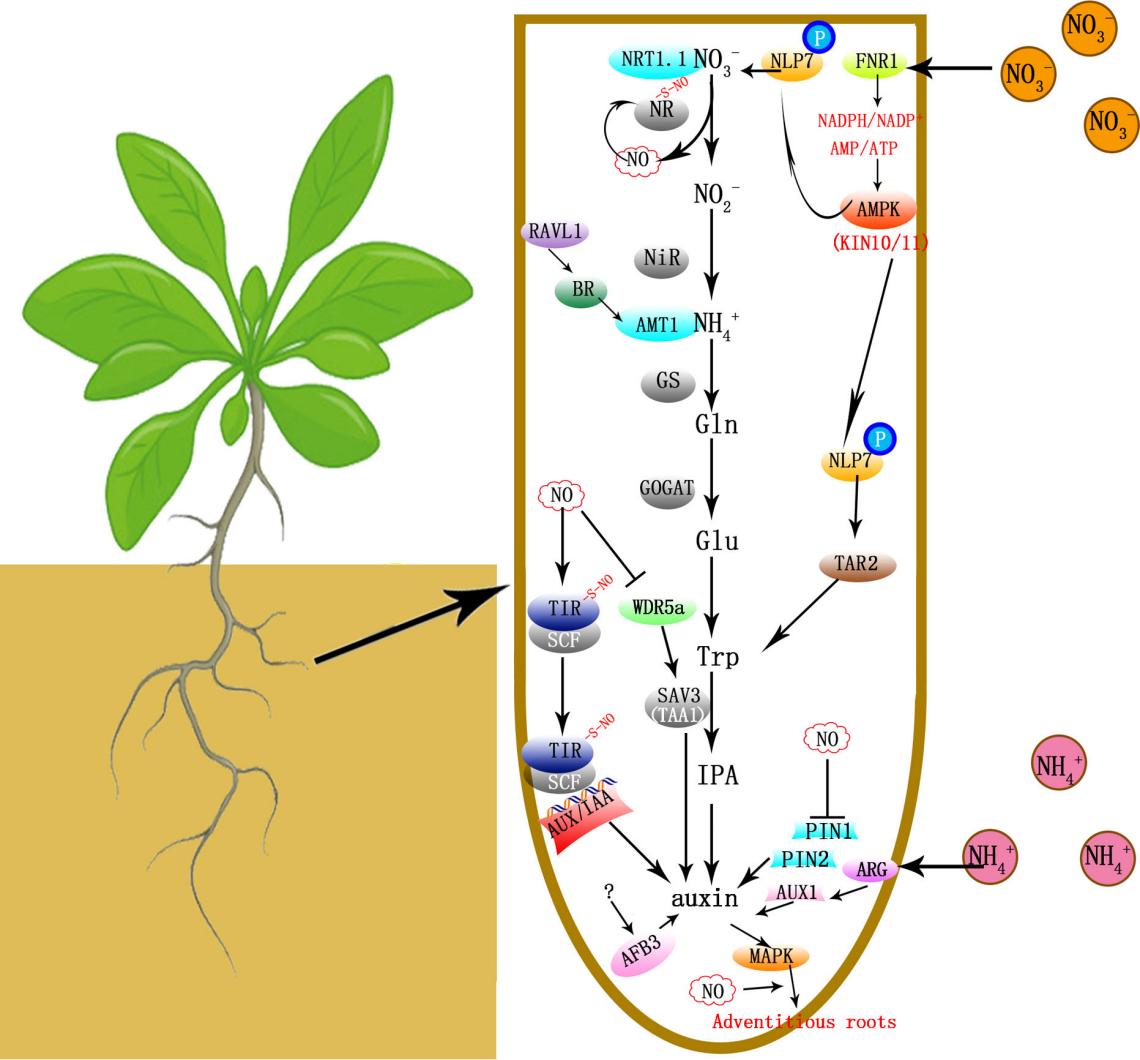
Metabolic pathways and signaling pathways that ${\text{NO}}_{3}^{-}$ , ${\text{NH}}_{4}^{+}$ , tryptophan and NO regulate auxin biosynthesis/signaling and plant growth/development. Nitrate reductase (NR) converts nitrate to nitrite. Then nitrite reductase (NiR) catalyzes the conversion of nitrite to ammonium, which is then converted to glutamate (Glu) *via* the glutamine (Gln) synthetase/glutamine oxoglutarate aminotransferase (GS/GOGAT) cycle and various amino acids, among which tryptophan is a key metabolite and the precursor of auxin. The nitrate transporter NRT1.1 is a nitrate transceptor controlling Abscisic Acid Responsive Element Binding Factor 3 (AFB3) expression. Nitrate inhibits Ferredoxin-NADP^+^-Oxidoreductase (*FNR1*) expression, therefore causing declines in NADPH/NADP^+^ and ATP/AMP ratios, which in-turn promotes adenosine monophosphate-activated protein kinase (AMPK; KIN10 and KIN11) activities and phosphorylates the NIN-like protein (NLP) transcription factor NLP7. NLP7 binds directly to the *TAR2* (Tryptophan Aminotransferase Related 2) promoter and activates auxin signaling. Some nitrate metabolite downstream of NR might affect *AFB3* transcription indirectly. NO is a by-product during NR functioning, however NO may deactivate NR by S-nitrosylation. When ${\text{NH}}_{4}^{+}$ is applied to shoots, ARG1 (Altered Response to Gravity 1) induces the auxin influx carrier AUX1 expression and basipetal auxin transport *via* PIN-Formed2 (PIN2) in root apices. And ${\text{NH}}_{4}^{+}$ mediated stresses were lessened when ABA signaling was activated (Li et al., 2012). And the brassinosteroid (BR) signaling transcription factor ABI3/VP1-Like 1 (RAVL1) controls BR-mediated activation of the ammonium transporter AMT1;2 and ${\text{NH}}_{4}^{+}$ absorption. The Tryptophan Aminotransferase of Arabidopsis 1 (TAA1; SAV3) catalyzes the synthesis of indole-3-pyruvic acid (IPA) from Trp and controls hypocotyl elongation and leaf expansion responsive to N changes. NO inhibits WD40-REPEAT 5a (WDR5a), which induces TAA1 (SAV3) expression. NO also increases the auxin receptor Transport Inhibitor Response 1 (TIR1) - the transcriptional repressor Auxin/Indole-3-Acetic Acid (Aux/IAA) protein interaction, which facilities the E3-ubiquitin ligase complex SFC-mediated AUX/IAA degradation and enhances the expression of auxin-regulated genes. The mitogen-activated protein kinase (MAPK) signaling cascade is activated during the adventitious root formation induced by auxin in a NO-mediated pathway. NO also inhibites acropetal auxin transport by lowering the abundance of PIN1.

Besides auxin, nitrate also regulates plant growth and development indirectly through interacting with cytokinin (Hu et al., 2020), ethylene (Zhou et al., 2022), abscisic acid (ABA; Sun et al., 2020), salicylic acid, gibberellins and brassinosteroids (Vega et al., 2019) (**Figure 1**).

## Roles of ${\text{NH}}_{4}^{+}$ on plant growth and development

Numerous studies have shown that the early genomic responses of rice and *Arabidopsis* to exogenous ${\text{NH}}_{4}^{+}$ result in a variety of distinct alterations in gene expression, metabolism, hormone signaling, redox state, and root system architecture (Lima et al., 2010; Patterson et al., 2010; Fernández-Crespo et al., 2015; Xuan et al., 2017; Xuan et al., 2019; Hachiya et al., 2021; Sun et al., 2021). Since many of these responses are not correlated with ${\text{NH}}_{4}^{+}$ assimilation rate directly, ${\text{NH}}_{4}^{+}$ has been suggested also as a signaling molecule; while the ammonium transporter AMT1 may serve as a sensor (Sonoda et al., 2003; Gaur et al., 2012). For example, AMT1;3 is necessary for ${\text{NH}}_{4}^{+}$ -dependent lateral root branching in *Arabidopsis* (Lima et al., 2010).

The ${\text{NH}}_{4}^{+}$ mediated suppression of root development is compromised in the auxin-resistant mutants *aux1*, *axr1*, and *axr2* (Cao et al., 1993). And the auxin influx carrier AUX1 inhibits lateral root emergence when ${\text{NH}}_{4}^{+}$ is applied to shoots (Li et al., 2011). The suppression of lateral root growth by ${\text{NH}}_{4}^{+}$ is related with ethylene generation in shoots (Li et al., 2013). Zou et al. (2013) interestingly found that, under NH4^+^ stress, *arg1* (Altered Response to Gravity 1) mutant displayed increased loss of root gravitropism. ARG1 is required for AUX1 protein expression and basipetal auxin transport *via* PIN-Formed2 (PIN2) in root apices. And ${\text{NH}}_{4}^{+}$ mediated stresses were lessened when ABA signaling was activated (Li et al., 2012). Recently, it was discovered that an important brassinosteroid (BR) signaling transcription factor ABI3/VP1-Like 1 (RAVL1) controls BR-mediated activation of AMT1;2 and ${\text{NH}}_{4}^{+}$ absorption in rice (Xuan et al., 2017) (**Figure 1**).

## Roles of nitrogen metabolite tryptophan on plant growth and development

Nitrogen in plants first assimilates ammonia to glutamate, which is then converted to various amino acids, among which tryptophan is a key metabolite and the precursor of auxin. Given that auxin plays a key role in plant growth and development, we focused on tryptophan (Trp) and auxin metabolism and signaling in this review. There are two auxin biosynthesis pathways, the Trp-dependent and the Trp-independent pathways; while the tryptophan-dependent pathway is the main one (Korasick et al., 2013; Ljung, 2013). Auxin is a potential mediator of N signaling that auxin content is inversely associated with N status in various plants (Tian et al., 2008; Kiba et al., 2011; Ma et al., 2014). Furthermore, jasmonic acid (JA) locally produced in response to mechanical wounding triggers the *de novo* formation of auxin through the induction of Trp-dependent pathways (Zhang G. et al., 2019; Pérez-Alonso et al., 2021).

A research revealed that tryptophan’s role as an auxin precursor on root elongation is rather straightforward (Jing et al., 2009). Besides the promotion on auxin biosynthesis, exogenous tryptophan increased root length and plant height and improved plant resistance to stresses by enhancing C/N metabolism and related enzyme activities (Mustafa et al., 2018).

After N treatments, plant leaves become thicker and narrower, and the chlorophyll level increases. Our previous study found that the changes in leaf thickness and width were largely compromised in the *shade avoidance 3* (*sav3*) mutant (Yang et al., 2022). The SAV3 protein catalyzes the synthesis of indole-3-pyruvic acid (IPA) from Trp, and is also named as Tryptophan Aminotransferase of Arabidopsis 1 (TAA1). SAV3 also controls hypocotyl elongation and leaf expansion under the shade condition (Tao et al., 2008), and regulates chlorophyll accumulation and nitrogen assimilation. Therefore SAV3 works as a master switch responsive to multiple environmental stimuli (Yang et al., 2022) (**Figure 1**).

## NO and auxin synergistically regulate plant growth and development

Although direct nitric oxide synthase (NOS) has not been found in higher plants, it has been suggested that NR’s main role is to provide nitrite, which in turn can be further reduced to NO. In other words, NO is a by-product during nitrate assimilation (Chamizo-Ampudia et al., 2017).

Our previous study indicated that the NR protein can be S-nitrosated by NO. The S-nitrosylation status of NR is negatively correlated with its enzymatic activity. Thus NO generated through NR catalysis may deactivate the enzyme itself by this S-nitrosylation-dependent negative-feedback regulation (Fu et al., 2018).

Nitric oxide (NO) is a multi-purpose gaseous signaling molecule (Domingos et al., 2015; Simontacchi et al., 2015). NO and auxin interact with each other in controlling root development (Correa-Aragunde et al., 2004; Fernández-Marcos et al., 2011; Jin et al., 2011; Chen and Kao, 2012; Sun et al., 2017; Basu et al., 2021) and root hairs formation (Lombardo et al., 2006). The mitogen-activated protein kinase (MAPK) signaling cascade is activated during the adventitious root formation induced by auxin in a NO-mediated but cGMP-independent pathway. The stimulation of MAPK has been proposed in modulating mitotic processes in root cells (Pagnussat et al., 2004; Hu et al., 2005).

Some other studies suggested that NO may also function in signaling pathways upstream of auxin (Terrile et al., 2012; Liu et al., 2018). NO lowers the level of auxin in the root apex by inhibiting WD40-REPEAT 5a (WDR5a), which induces TAA1 (SAV3) and auxin accumulation (Liu et al., 2018). But NO increases the auxin receptor Transport Inhibitor Response 1 (TIR1) and the transcriptional repressor Auxin/Indole-3-Acetic Acid (Aux/IAA) protein interaction *via* S-nitrosylation on TIR1, which enhances the expression of auxin-regulated genes in the whole root (Terrile et al., 2012).

Additionally, NO-overproducing mutants and pharmacological treatments showed that, at high concentrations, NO inhibited acropetal auxin transport in Arabidopsis roots by lowering the abundance of the auxin efflux protein PIN1 through a proteasome-independent post-transcriptional mechanism (Fernández-Marcos et al., 2011).

Our previous study demonstrated that NO dramatically decreased monosaccharide catabolism by inhibiting sugar metabolic enzymes *via* S-nitrosylation. As a result, NO treatments reduced starch granule formation in root tips and compromised root gravitropism indirectly (Zhang et al., 2017).

Besides these putative mechanisms of NO on auxin transport and signaling, NO also regulates plant growth and development indirectly through interacting with ethylene (Du et al., 2014), cytokinin (Feng et al., 2013; Liu et al., 2013), ABA (Sang et al., 2008; Wang et al., 2015), gibberellin and light signaling (Bai et al., 2014).

Both nitrogen and NO treatments postpone plant flowering (He et al., 2004; Yuan et al., 2016). However, the high nitrogen condition reduced the amplitudes of transcripts of all circadian clock genes (Yuan et al., 2016). While NO enhanced the amplitudes of central oscillators, but reduced the amplitudes of circadian-clock output genes, *GI* (*GIGANTEA*) and *CO* (*CONSTANS*). NO induced S-nitrosation modification on GI and CO proteins, but not on the other circadian clock proteins (Zhang Z. W. et al., 2019). Thus nitrogen and NO rely on overlapping but different signaling pathways to regulate plant flowering (**Figure 1**).

## Conclusions and perspectives

Nitrogen and its metabolites regulate plant growth and development through multiple and complex mechanisms. Nitrate assimilation metabolites, ${\text{NO}}_{3}^{-}$ , ${\text{NH}}_{4}^{+}$ , tryptophan and the by-product NO, as well as the key enzymes NR and TAA1 are all involved, with interacting with phytohormone signals. Interestingly, NO may deactivate NR by S-nitrosylation (Fu et al., 2018). Whether NO also generates a feedback regulation on TAA1 requires further studies.

It is interesting to note that the *AFB3* transcript did not decrease in the NR-deficient mutant after 1 hour of nitrate treatment. Thus some nitrate metabolite downstream of NR might affect *AFB3* transcription indirectly (Vidal et al., 2010; Vidal et al., 2013). Which metabolite plays the key role needs further investigations. Both nitrate and NO repress auxin accumulation by decreasing TAA1 expression (Liu et al., 2018; Yang et al., 2022). Whether TAA1 works upstream of AFB3 requires further explorations. And we also don’t know whether TAA1 activity is associated with the cellular tryptophan level.

Both ammonium poisoning and NO accumulation increase loss of root gravitropism and inhibit root elongation (Zou et al., 2013; Zhang et al., 2017). The crosstalk between ${\text{NH}}_{4}^{+}$ signaling and NO metabolism in root morphogenesis would also be an interesting research direction.

## Author contributions

SY conceived the project. Y-FF, X-YY, and Z-WZ. performed the literature search. Y-FF and SY wrote the manuscript with input from X-YY and Z-WZ. All authors contributed to the article and approved the submitted version.
